# Association between gut microbiota and sensorineural hearing loss: a Mendelian randomization study

**DOI:** 10.3389/fmicb.2023.1230125

**Published:** 2023-10-17

**Authors:** Qiuyuan Yin, Guolin Shi, Lei Zhu

**Affiliations:** ^1^State Key Laboratory for Conservation and Utilization of Bio-Resources in Yunnan, School of Life Sciences, Yunnan University, Kunming, Yunnan, China; ^2^Department of Neurosurgery, The Second Affiliated Hospital of Kunming Medical University, Kunming, Yunnan, China

**Keywords:** sensorineural hearing loss, gut microbiota, gut–inner ear axis, Mendelian randomization study, causal effect

## Abstract

**Background:**

Several recent studies speculated that the gut microbiota is associated with sensorineural hearing loss (SNHL) and proposed the concept of the gut–inner ear axis. However, the causal effect of gut microbiota on SNHL is still unknown. In this study, we performed a two-sample Mendelian randomization (MR) analysis to estimate the causal effect of gut microbiota on SNHL.

**Methods:**

Gut microbiota data were obtained from the largest available genome-wide association study (*n* = 18,340) conducted by the MiBioGen consortium. The summary statistics of SNHL were obtained from the FinnGen consortium R8 release data (28,310 cases and 302,750 controls). The causal effects were estimated with inverse-variance weighted, MR-Egger, and weighted median. Reverse Mendelian randomization analysis was performed on the bacteria that were found to be associated with SNHL in forward Mendelian randomization analysis. We then performed sensitivity analyses, including Cochran's *Q-*test, MR-Egger intercept test, MR-PRESSO, cML-MA-BIC, and leave-one-out analysis, to detect heterogeneity and pleiotropy.

**Results:**

The inverse-variance weighted results suggested that *Lachnospiraceae (UCG001)* had a significant protective effect against SNHL (odds ratio = 0.85, 95% confidence interval: 0.78–0.93, *P* = 6.99 × 10^−4^). In addition, *Intestinimonas* (odds ratio = 0.89, 95% confidence interval: 0.82–0.97, *P* = 8.53 × 10^−3^) presented a suggestively protective effect on SNHL. *Rikenellaceae (RC9gutgroup)* (odds ratio = 1.08, 95% confidence interval: 1.02–1.15, *P* = 0.01) and *Eubacterium (hallii group)* (odds ratio = 1.12, 95% confidence interval: 1.00–1.24, *P* = 0.048) suggestively increase the risk of SNHL. The results of the reverse MR analysis showed that there is no significant causal effect of SNHL on the gut microbiota. No significant heterogeneity of instrumental variables or pleiotropy was detected.

**Conclusion:**

The evidence that the four genera mentioned above are associated with SNHL supports the hypothesis of a gut–inner ear axis. Our study provides microbial markers for the prevention and treatment of SNHL, and further studies are needed to explore the mechanisms of the gut microbiome–inner ear axis in health and diseases.

## Background

Hearing loss interferes with the quality of life. More than 1.5 billion people worldwide experience some degree of hearing loss during their lifetime, and at least 430 million of them need additional care. The World Health Organization (2021) estimates that this number will increase to 2.5 billion by 2050. Sensorineural hearing loss (SNHL) is the most common type and accounts for the majority of all hearing loss, which is characterized by irreversible damage to cochlear hair cells, auditory nerves, or the central nervous system (Tanna et al., 2020). SNHL is also associated with dementia, Alzheimer's disease, and other cognitive disorders (Hung et al., 2015; Fortunato et al., 2016; Shen et al., 2021). Although the pathogenesis of SNHL is still not fully understood, a variety of causes, including genetic factors, aging, chronic noise exposure, ototoxic medications, and head injury, are believed to be involved (Chau et al., 2010; Kuhn et al., 2011; Tarshish et al., 2013). Moreover, obesity and a high-fat diet have also been linked to hearing loss (Tang et al., 2019; Kociszewska et al., 2021).

The diversity and composition of the gut microbiota have been observed to change significantly after chronic noise exposure (Cui et al., 2018). SNHL has also been reported to be the most common inner ear disorder that positively correlates with gastrointestinal (GI) conditions such as inflammatory bowel diseases (IBDs) and celiac disease (CD) (Karmody et al., 2009; Kalyoncu et al., 2010; Wengrower et al., 2016; Fousekis et al., 2018). In addition, histological studies have elucidated the analogous structures and functions between the blood–labyrinth barrier (BLB), a key regulator in inner ear endolymph and perilymph ionic concentration, and the blood–brain barrier (BBB), which represents an important interface in mediating the gut–brain axis (Banks, 2006; Hirose et al., 2014; Kociszewska et al., 2021; Song et al., 2021). All information provided by these studies implied the existence of the gut–inner ear axis; however, no studies found a causality between gut microbiota and SNHL. Owing to the inherent defects of conventional designs, previous observational studies are unable to entirely exclude the possibility of reverse causality and confounding factors, which could potentially result in biased associations and conclusions (Sekula et al., 2016). Moreover, microbiota-related observational studies are susceptible to confounding factors such as age, environment, dietary patterns, and lifestyle (Rinninella et al., 2019). These conditions limit the reliability of causality between gut microbiota and SNHL to some extent.

Mendelian randomization (MR) is increasingly applied to infer credible causal relationships between risk factors and disease outcomes (Richmond and George Davey Smith, 2022). Based on the random assortment of genetic variants during meiosis, MR used environmental exposure-related genetic variations as instrumental variables (IVs) to assess the association between exposures and outcomes (Burgess and Thompson, 2015). Since genetic variants are randomly assigned at conception before disease onset, the results of MR analysis are not affected by confounding factors (Davey Smith, 2014). MR has been widely used to explore the causality between gut microbiota and diseases, e.g., optic neuritis (Liu et al., 2022), preeclampsia-eclampsia (Li et al., 2022), delirium (Yu et al., 2023), smoking (Fan et al., 2023), metabolic diseases (Sanna et al., 2019), and autoimmune diseases (Xu et al., 2022).

In this study, we applied the MR design to evaluate the potential causal effect of gut microbiota on SNHL. Our study is the first to demonstrate the existence of the gut–inner ear axis in a causal way and will provide valuable suggestions for preventive intervention strategies for SNHL.

## Methods

### Study design

**Figure 1:**
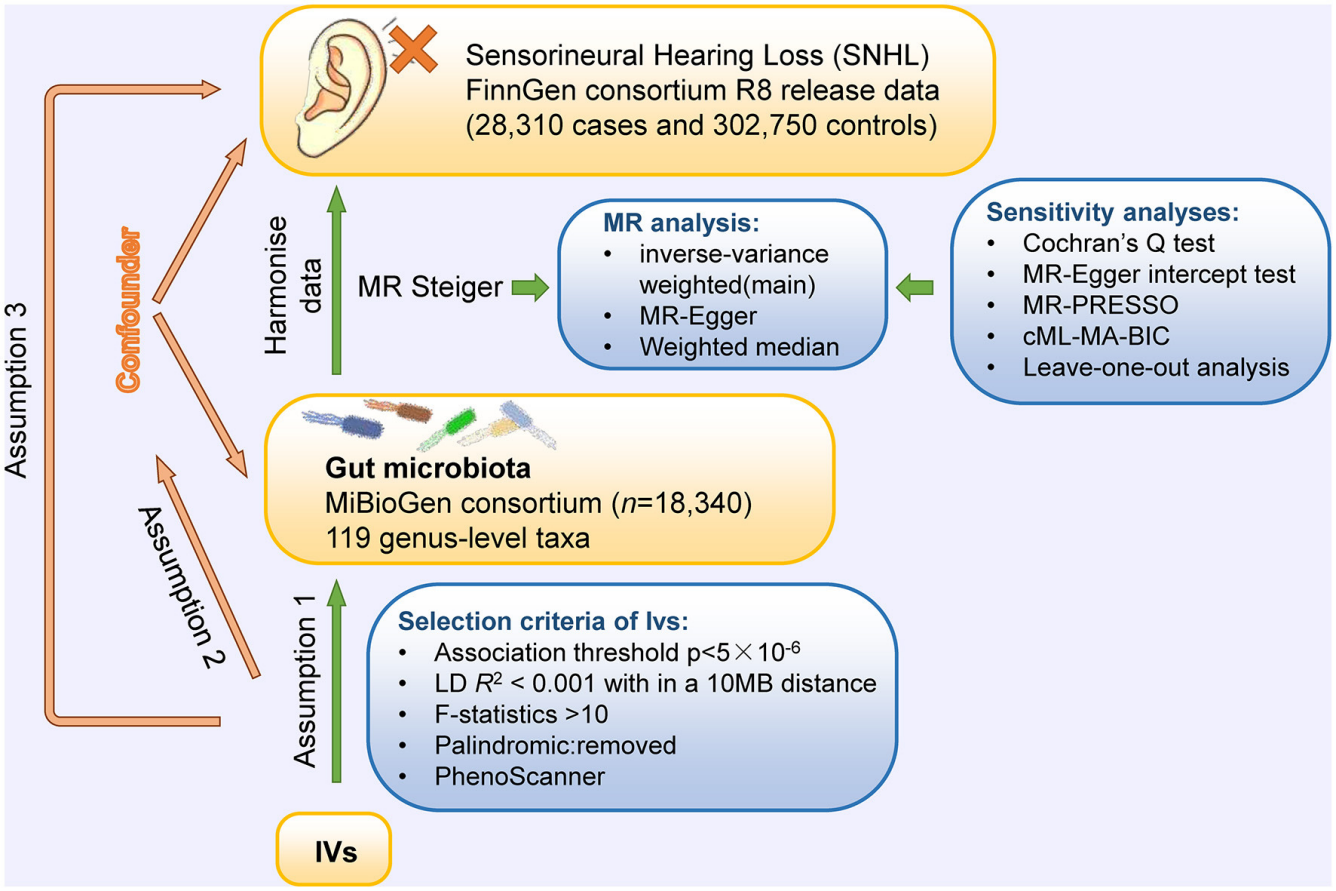
Conceptual framework for the Mendelian randomization analysis of causal effects of gut microbiota on the risk of SNHL. Assumption 1, genetic variants are robustly associated with exposure; assumption 2, genetic variants are not associated with potential confounders; and assumption 3, genetic variants affect outcomes only through the exposure of interest.

To explore the causal relationships of gut microbiota on SNHL, a two-sample MR was performed using instrumental variables (IVs) extracted from the largest investigation of the genetics of the gut microbiome (Kurilshikov et al., 2021). To avoid sample overlap, GWAS summary data of SNHL were obtained from the FinnGen Project (Kurki et al., 2022). The MR design is based on three assumptions: (1) genetic variants are robustly associated with exposure data; (2) genetic variants are not associated with potential confounders; and (3) genetic variants affect the outcome only through the exposure of interest (Boef et al., 2015). In this study, multiple methods were used for MR and sensitivity analyses to confirm the robustness of our results. The conceptual MR framework is presented in Figure 1. We reported the MR study following the recommendations of the STROBE-MR Guidelines (Supplementary STROBE-MR Checklist).

### Data source

The summary statistics of gut microbiota were obtained from the MiBioGen consortium (Kurilshikov et al., 2021). This is currently the largest genome-wide meta-analysis of the human microbiome, containing a total of 18,340 samples of 16S rRNA gene sequencing data from 24 population cohorts. Most of them are of European descent (*n* = 13,266). The microbial composition was profiled by targeting three distinct variable regions of the 16S rRNA gene: V4, V3–V4, and V1–V2, and conducting taxonomic classification using direct taxonomic binning. The microbiota quantitative trait loci (mbQTL) mapping analysis was performed to identify host genetic variants that are associated with the abundance of gut bacterial taxa (Kurilshikov et al., 2021). The genus had the lowest taxonomic level in this study. In addition to the 12 unknown genera, 119 genus-level taxa were included in the current study for MR analysis. GWAS summary statistics for SNHL were obtained from the FinnGen consortium R8 release data. This GWAS contained 28,310 cases and 302,750 controls. The first 10 principal components and genotyping batch were corrected during the analysis (Kurki et al., 2022).

To investigate the potential mechanisms by which genetically proxied genera affect the risk of SNHL, a two-step MR was performed to calculate the mediating effect. Mediating factors include BMI, triglycerides, total cholesterol, low-density lipoprotein (LDL), high-density lipoprotein (HDL), type 1 diabetes, and type 2 diabetes. To avoid overlapping with the exposures and outcomes, the summary-level results of these potential mediators were retrieved from the UK Biobank.

### Selection criteria of instrumental variables

As per the three assumptions stated in the design of this study, quality control was performed on single-nucleotide polymorphisms (SNPs) to make our results robust. Similar to most current MR studies, the genome-wide significance threshold (*P* < 5 × 10^−8^) was selected to screen SNPs. Because of the limited number of SNPs meeting genome-wide significance, we used SNPs with a more relaxed threshold (*P* < 5 × 10^−6^) as potential IVs of each genus. To ensure independence among IVs, we applied linkage disequilibrium clumping with a clumping window of 10 MB and *R*^2^ < 0.001 based on European ancestry reference data from the 1,000 Genomes Project. Meanwhile, to avoid bias owing to the employment of weak instruments, F-statistics were calculated for each SNP to measure the statistical strength, and only strong IVs (F-statistics > 10) for each of our exposures of interest remained. Ambiguous and palindromic SNPs of which the effect cannot be corrected in the harmonizing process were excluded. Since MR frequently generates false positives in the presence of genetic correlation between traits (O'Connor and Price, 2018; Reay et al., 2022), the SNPs associated with the outcome (SNHL) were removed. In reverse MR analysis, the genome-wide significance threshold of exposure data (SNHL) was set to *P* < 5 × 10^−8^; the remaining criteria and parameters are consistent with forward MR.

We also scanned with the PhenoScanner V2 (www.phenoscanner.medschl.cam.ac.uk), a database of human genotype–phenotype associations, to detect whether these IVs were associated with the potential risk factors, including obesity and diabetes (Tanna et al., 2020), and remove SNPs associated with any of these potential confounders.

### Mendelian randomization analyses

Three different methods of MR, random-effect inverse-variance weighted (IVW), MR-Egger, and weighted median, were performed to estimate the causal effect of gut microbiota on SNHL. IVW estimates were used as the main analysis, which combined the Wald ratio of each SNP on the outcome and obtained a pooled causal estimate. If horizontal pleiotropy was not present, the IVW results would be unbiased (Burgess et al., 2016). Meanwhile, MR-Egger and weighted median were used to improve the IVW estimates as they could provide more robust estimates in a broader set of scenarios, despite being less efficient (wider confidence interval). MR-Egger allows all genetic variants to have a pleiotropic effect but requires that the pleiotropic effects be independent of the variant-exposure association (Bowden et al., 2015). The weighted median method allows for the correct estimation of causal association when up to 50% of instrumental variables are invalid (Hartwig et al., 2017).

### Sensitivity analysis

Sensitivity analysis has been performed to detect the underlying pleiotropy and heterogeneity because they can seriously affect MR estimates. Cochran's *Q-*test was applied to detect heterogeneity (Del Greco et al., 2015). There was no heterogeneity detected if the *p-*value of Cochran's *Q*-test was >0.05. The pleiotropic analysis was preliminarily judged by the intercept of the MR-Egger regression (*P* < 0.05 was considered possible pleiotropy in IVs) (Burgess and Thompson, 2017). MR-Pleiotropy Residual Sum and Outlier Methods (MR-PRESSO) were also used to assess and correct horizontal pleiotropy (Ong and MacGregor, 2019). Meanwhile, a leave-one-out analysis was performed to evaluate whether the MR estimate was driven or biased by an SNP. We also used a constrained maximum likelihood and model averaging-based MR method, called cML-MA, to control correlated and uncorrelated pleiotropic effects in this study (Xue et al., 2021).

### Statistical analysis

*F*-statistic was used to calculate the strength of IVs using the formula $F=\frac{R^{2}\times \left(N-1-K\right)}{\left(1-R^{2}\right)\times K}$, where *R*^2^ represents the proportion of variance in the exposure explained by the genetic variants, *N* represents the sample size, and *K* represents the number of IVs (Staiger and Stock, 1994). In addition, the website (http://cnsgenomics.com/shiny/mRnd/) was used to calculate the power (Brion et al., 2013).

To account for multiple testing in our primary analyses, false discovery rate (FDR) correction was performed by applying the *q*-value procedure, with a false discovery rate of *q*-value < 0.1 (Storey and Tibshirani, 2003). The genera of gut microbiota and SNHL were considered to have a suggestive association when *P* < 0.05 but *q* ≥ 0.1.

All the analyses were performed by the two-sample MR package (version 0.5.6) (Hemani et al., 2017), MRcML package (Xue et al., 2021), and qvalue package (version 2.15.0) (Storey and Tibshirani, 2003) of the R program (version 4.2.1).

## Results

**Figure 2:**
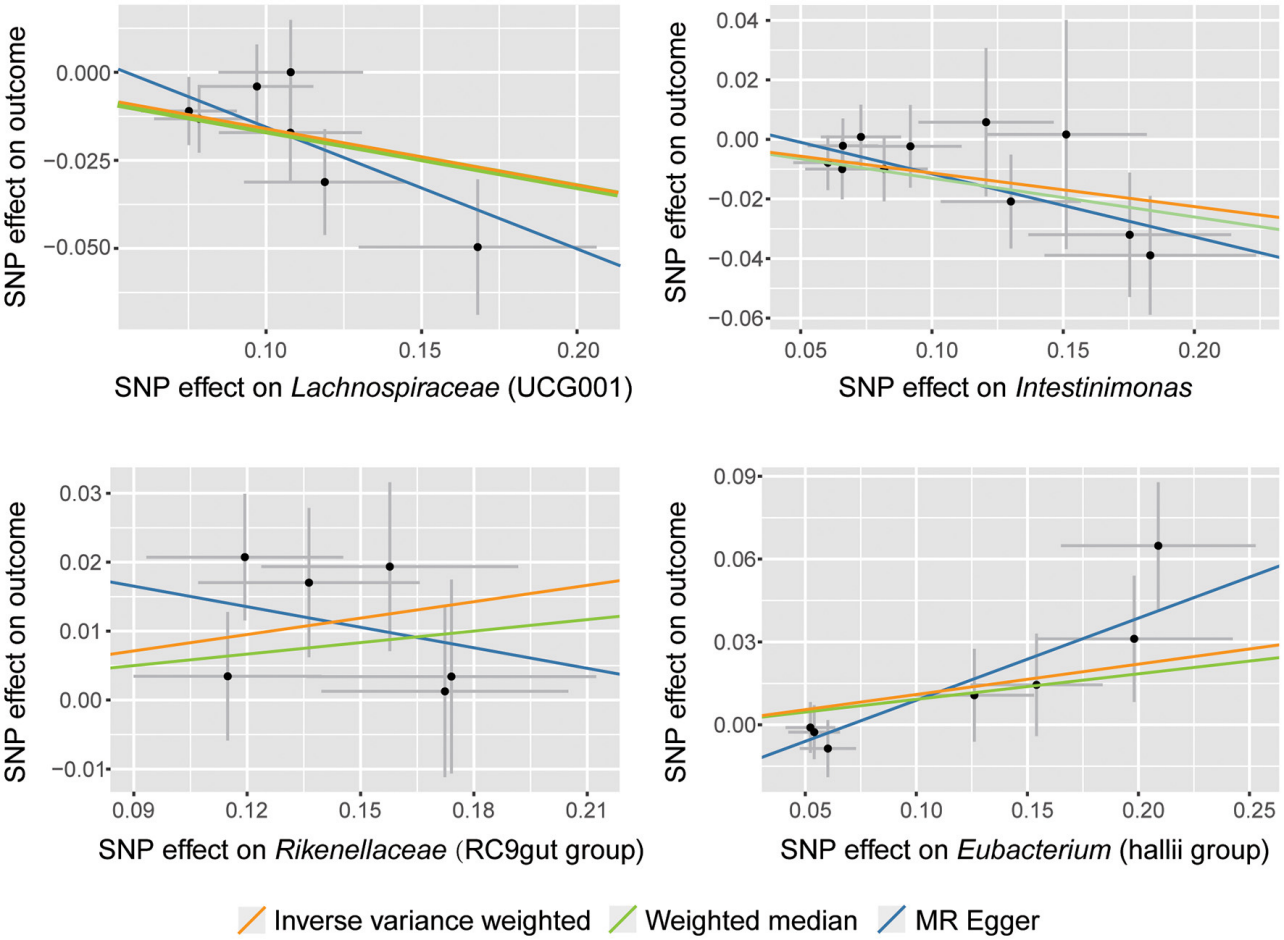
Scatter plots for the causal association between gut microbiota and SNHL.

According to the selection criteria for IVs, a total of 661 SNPs were used as IVs for 119 bacterial genera (Supplementary Table 1). As shown in Table 1, Supplementary Table 2, and Figure 2, four bacterial genera were found to be associated with SNHL by IVW results. *Lachnospiraceae (UCG001)* had a significant protective effect against SNHL (IVW: OR = 0.85, 95% CI: 0.78–0.93, *P* = 6.99 × 10^−4^, *q* = 0.08) with sufficient power (0.91). Meanwhile, similar estimates were obtained using the MR-Egger regression (OR = 0.71, 95% CI = 0.48–1.04, *p* = 0.14, *q* = 1.00) and weighted median approaches (OR = 0.85, 95% CI = 0.75–0.97, *p* = 0.016, *q* = 1.00), though the association was not statistically significant. In addition, *Intestinimonas* presented a suggestively protective effect on SNHL (IVW: OR = 0.89, 95% CI: 0.82–0.97, *P* = 8.53 × 10^−3^). In addition to the protective effect, *Rikenellaceae (RC9gutgroup)* (IVW: OR = 1.08, 95% CI: 1.02–1.15, *P* = 0.01) and *Eubacterium (hallii group)* (IVW: OR = 1.12, 95% CI: 1.00–1.24, *P* = 0.048) suggestively increase the risk of SNHL.

**Table 1:** MR estimates for the association between gut microbiota and SNHL.

**Bacterial taxa (exposure)**	**MR method**	**No. of SNP**	**OR**	**95%CI**	***p*-value**	***q*-value**	**Power**
Lachnospiraceae (UCG001)	IVW	7	0.85	0.78–0.93	6.99E-04	0.08	0.91
	MR-Egger	7	0.71	0.48–1.04	0.140	1.00	
	Weighted median	7	0.85	0.75–0.97	0.016	1.00	
Intestinimonas	IVW	11	0.89	0.82–0.97	8.53E-03	0.49	0.85
	MR-Egger	11	0.81	0.64–1.02	0.105	1.00	
	Weighted median	11	0.88	0.78–0.98	0.024	1.00	
Rikenellaceae (RC9gutgroup)	IVW	6	1.08	1.02–1.15	0.013	0.49	0.74
	MR-Egger	6	0.91	0.62–1.32	0.633	1.00	
	Weighted median	6	1.06	0.97–1.15	0.188	1.00	
Eubacterium (hallii group)	IVW	7	1.12	1.00–1.24	0.048	0.99	0.79
	MR-Egger	7	1.35	1.11–1.64	0.030	1.00	
	Weighted median	7	1.10	0.95–1.26	0.201	1.00	

MR, Mendelian randomization; SNHL, sensorineural hearing loss; SNP, single-nucleotide polymorphism; OR, odds ratio; CI, confidence interval; IVW, inverse-variance weighted. Power was calculated by OR of IVW at the website http://cnsgenomics.com/shiny/mRnd/.

Since all IVs used in the four causal associations have *F*-statistics > 10, there is no bias for weak IVs in our results (Supplementary Table 1). The results of Cochran's IVW *Q-*test showed no significant heterogeneity of these IVs (*P* > 0.05, Supplementary Table 3). In addition, there was no significant directional horizontal pleiotropy according to the results of the MR-Egger regression intercept analysis (*P* > 0.05, Supplementary Table 4).

**Figure 3:**
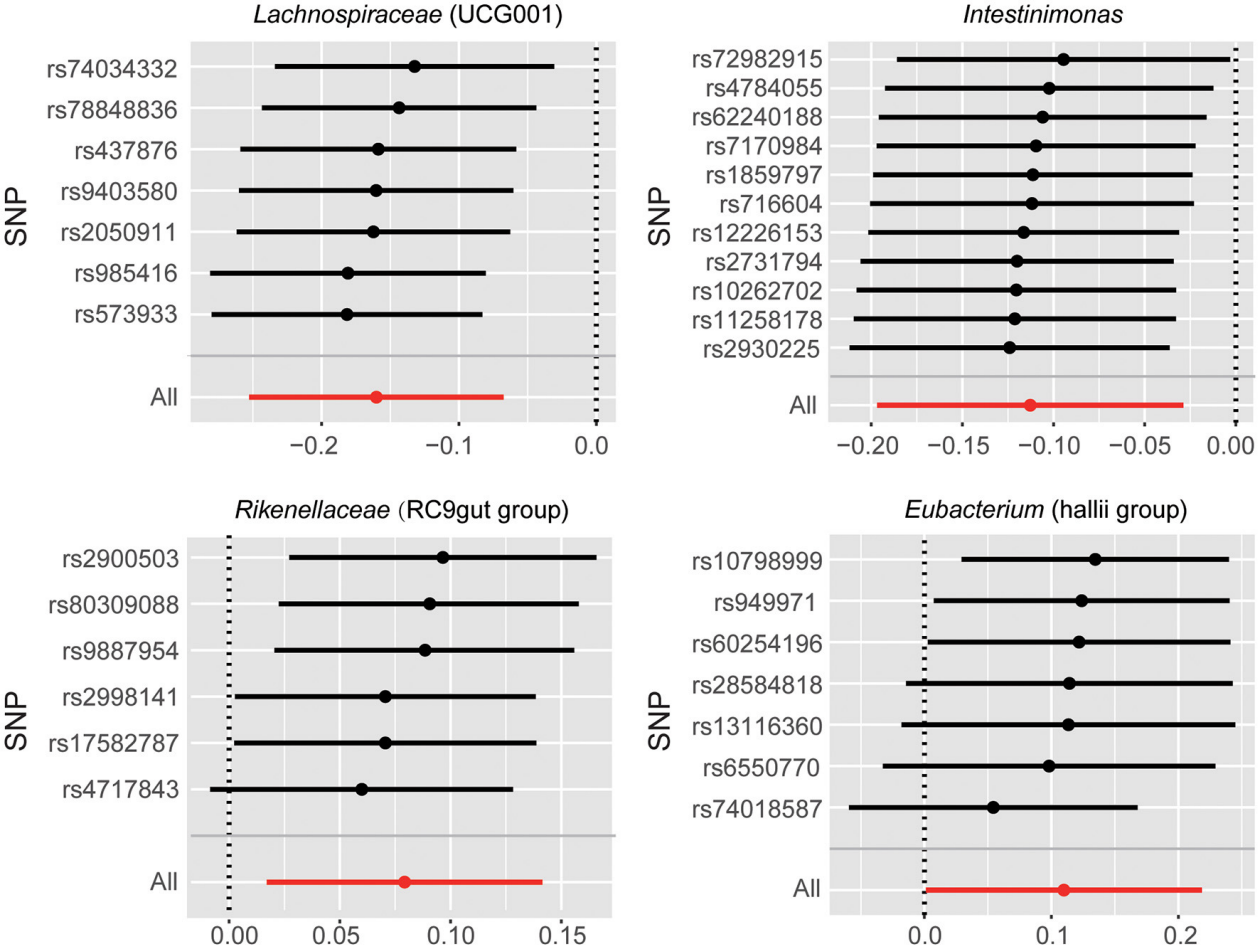
Leave-one-out plots for the causal association between gut microbiota and SNHL.

There were potential outliers of the IVs of *Eubacterium (hallii group)* that were present on visual inspection in leave-one-out plots (Figure 3). However, further MR-PRESSO analysis did not detect any significant outlier SNPs (global test *P* > 0.05, Supplementary Table 5). Therefore, there was insufficient evidence for horizontal pleiotropy in the association between these bacteria and SNHL.

To further control correlated and uncorrelated pleiotropic effects in this study, the cML-MA-BIC method was used to recalculate the MR results of the four bacterial genera. The results of cML-MA-BIC were consistent with IVW (Supplementary Table 6), which suggested our results were robust after considering the associated pleiotropy.

According to the results of reverse MR analysis, there was a suggestive association between SNHL and *Rikenellaceae (RC9gutgroup)* (IVW: OR = 1.26, 95% CI: 1.02–1.55, *P* = 0.029); however, such association became insignificant after correction for FDR (*q* = 0.12). No significant causal association was found between SNHL and the other three bacterial genera (Supplementary Tables 7, 8). Results of Cochran's IVW *Q-*test showed that there was no significant heterogeneity in IVs of SNHL (Supplementary Table 9). MR-Egger regression intercepted item analysis (Supplementary Table 10), and MR-PRESSO analysis also did not detect significant horizontal pleiotropy (Supplementary Table 11).

To investigate the mediators of the effect of the above four bacteria genera on SNHL risk, a two-step MR was conducted to calculate the mediating effect. BMI, triglycerides, total cholesterol, LDL, HDL, type 1 diabetes, and type 2 diabetes were included as mediating factors. However, we identify no mediator that is influenced by the four bacteria genera and has a causal effect on SNHL risk at the same time (Supplementary Tables 12, 13), suggesting that the four bacteria genera may not affect SNHL through the above mediating factors.

## Discussion

In this study, we performed a two-sample MR analysis to evaluate the causal association between gut microbiota and SNHL, based on the summary statistics of gut microbiota from the largest GWAS meta-analysis conducted by the MiBioGen consortium and the summary statistics of SNHL from the FinnGen consortium R8 release data. We found that *Lachnospiraceae (UCG001)* had significant protective effects on SNHL, and three genera of gut microbiota had suggestive protective effects (*Intestinimonas*) or harmful effects [*Rikenellaceae (RC9gutgroup)* and *Eubacterium (hallii group)*] against SNHL. Our study is the first to demonstrate the existence of the gut–inner ear axis with causal evidence.

Previous studies proved that gut microbiota can affect the permeability of the blood–brain barrier (BBB), which is pivotal to brain development and function (Braniste et al., 2014). Interestingly, there is a similar organization in the inner ear called the blood–labyrinth barrier (BLB), which owns the analogous structures and functions with BBB and plays an important role in inner ear fluid homeostasis (Juhn et al., 2001). Changes in the gut microbiota can lead to systemic inflammation affecting multiple organ systems, including the brain and the inner ear (Kociszewska and Vlajkovic, 2022a). This inflammatory response increases the permeability of BLB (Ichimiya et al., 2000). Recent studies postulate that the microbial metabolites and pathogens released from the gut increase BLB permeability, which allows the spreading of inflammatory processes to the inner ear, leading to hearing dysfunctions (Denton et al., 2022; Kociszewska and Vlajkovic, 2022b). The microbes we found in this study, *Lachnospiraceae (UCG001)*, can produce acetic acid, one kind of short-chain fatty acid (SCFA) (Guo and Li, 2019) that has anti-inflammatory and immunomodulatory properties (Dalile et al., 2019). *Intestinimonas* have been reported to play a crucial role in promoting the metabolism of lysine to butyric acid, which also belongs to SCFAs and has anti-inflammatory effects (Bui et al., 2016, 2020). Previous studies found that *Rikenellaceae (RC9gutgroup)* displayed a strong positive association with obesity and inflammation (Sun et al., 2019; Ahmad et al., 2020; Ma et al., 2020). Therefore, we assumed that proinflammatory cytokines generated from gut microbiota, such as *Rikenellaceae (RC9gutgroup)*, can lead to cochlear damage via their impact on BLB permeability. On the other hand, anti-inflammatory cytokines, such as SCFAs produced by *Lachnospiraceae (UCG001)* and *Intestinimonas*, can protect the cochlea from inflammation damage.

Puzzlingly, *Eubacterium (hallii group)*, which had been identified as a candidate for the next-generation probiotics category with great potential to avert inflammatory disorders (Almeida et al., 2020), has been identified as a suggestive risk factor for SNHL in this study. A recent study reported that *Eubacterium (hallii group)* was enriched in type 1 diabetes mellitus (T1DM) patients and displayed a positive correlation with fasting blood glucose (Liu et al., 2021). Meanwhile, patients with T1DM have a significantly greater prevalence of hearing loss compared to healthy controls (Mujica-Mota et al., 2018). However, more mechanism studies are needed to reveal the relationship between *Eubacterium (hallii group)* and SNHL.

In this study, concordant directions and similar magnitudes across various MR models confirmed the robustness of our MR results. However, the MR estimates of MR-Egger and IVW were inconsistent in *Rikenellaceae (RC9gutgroup)* (Figure 2). According to previous studies, we could tighten the instrument *p-*value threshold (Ong and MacGregor, 2019). However, the instruments' *p-*value for *Rikenellaceae (RC9gutgroup)* was almost the same, so we cannot tighten the *p-*value. Finally, we accepted the results of IVW and identified *Rikenellaceae (RC9gutgroup)* as a risk factor for SNHL according to the following reasons: First, our sensitivity analysis showed no significant heterogeneity or pleiotropy was detected; the inverse-variance weighted (IVW) method is the most powerful method in this situation (Burgess et al., 2013; Lin et al., 2021). Second, the results of the weighted median (Table 1), MR-PRESSO (Supplementary Table 5), and cML-MA-BIC (Supplementary Table 6) were consistent with IVW. Third, *Rikenellaceae (RC9gutgroup)* had been reported to display a strong positive association with inflammation (Sun et al., 2019; Ahmad et al., 2020; Ma et al., 2020), which could lead to increased BLB permeability and even hearing loss. Even so, the causal effect of *Rikenellaceae (RC9gutgroup)* on SNHL should be taken cautiously, and further verification is required.

Similar to the gut–brain axis, recent studies have speculated that there is bidirectional communication between the gut microbiome and the inner ear (Denton et al., 2022). We then performed reverse MR to estimate the causal association between SNHL and gut microbiota. However, our results fail to support the hypothesis. The reverse causal association could not be completely excluded since the sample size of the gut microbiota is relatively small compared to the outcome dataset. The accuracy of MR will be affected to some extent.

Previous studies speculated that obesity and diabetes may relate to SNHL. However, our four bacterial genera did not affect SNHL through these mediators. Further studies are needed to explore the mechanisms of the effect between the four bacterial genera and SNHL. In addition, we found that triglycerides significantly increase the risk of SNHL (IVW: OR = 1.08, 95% CI: 1.02–1.14, *P* = 0.005), which deserves further research.

The strengths of our study are manifested in many ways. First, MR analysis can simulate randomized controlled trials in observational settings, which are widely accepted in causal research. Compared to the observational study, using an MR design, our study is largely free from reverse causation and residual confounding. In addition, the GWAS summary data used in this study were obtained from the largest scale of meta-studies to date, ensuring the strength of the instruments in the MR analysis. Third, various methods were used in sensitivity analysis, and no significant heterogeneity of instrumental variables or pleiotropy was detected. To make our MR results robust, non-overlapping exposure and outcome summary-level data were used to avoid bias. To the best of our knowledge, this is the first study that has performed an MR analysis to address the causal relationship between gut microbiota and SNHL. Our study supports the existence of the gut–inner ear axis in a causal way and provides new biomarkers for the prevention and treatment of SNHL.

However, several limitations should be noted in our study while interpreting the results. First, there are different types of SNHL, such as presbycusis, noise-induced hearing loss, and others. Detailed subgroup analyses were unable to be performed since summary statistics rather than raw data were used in the analysis. Second, since the lowest taxonomic data in the exposure dataset we can acquire is genus level, we cannot estimate the causal association between species and strain level. The MR results of a genus are contributed by all the species and strains in the genus, which is probably the reason why the OR value is relatively close to 1. Third, since the limited number of SNPs reached genome-wide significance (*P* < 5 × 10^−8^), we thus relaxed the *P* threshold. Following this adjustment, we used the FDR correction to restrict the possibility of false positives. The last aspect that should be discussed is that the majority of the participants in this study are European. Although population heterogeneity will be largely avoided, the results of our study may not be entirely applicable to subjects from other populations. In addition, experimental models are needed to further verify the relationship between gut microbiota and hearing loss in future.

## Conclusion

In summary, our study suggested that gut microbiota influence the risk of SNHL, providing the latest evidence about the existence of the gut–inner ear axis. We found that *Lachnospiraceae (UCG001)* was associated with SNHL. Further studies are needed to illustrate the protective effect and mechanism of probiotics on SNHL. In addition, although reverse MR estimates did not support the causal association of SNHL with gut microbiota, it cannot be ruled out the possibility that SNHL may affect the gut microbial ecosystem. Further studies are needed to confirm these reverse MR estimates.

## Data availability statement

The original contributions presented in the study are included in the article/Supplementary material, further inquiries can be directed to the corresponding author.

## Ethics statement

This research has been conducted using published studies and consortia providing publicly available summary statistics. All original studies have been approved by the corresponding ethical review board, and the participants have provided informed consent. In addition, no individual-level data were used in this study. Therefore, no new ethical review board approval was required.

## Author contributions

QY and LZ designed the study. QY, GS, and LZ analyzed and interpreted the data, drafted, and revised the manuscript. All authors contributed to the article and approved the submitted version.

## References

[B1] Ahmad M. I. Ijaz M. U. Hussain M. Haq I. U. Zhao D. Li C. (2020). High-fat proteins drive dynamic changes in gut microbiota, hepatic metabolome, and endotoxemia-TLR-4-NFκB-mediated inflammation in mice. J. Agric. Food Chem. 68, 11710–11725. 10.1021/acs.jafc.0c02570 33034193

[B2] Almeida D. Machado D. Andrade J. C. Mendo S. Gomes A. M. Freitas A. C. (2020). Evolving trends in next-generation probiotics: a 5W1H perspective. Crit Rev. Food Sci. Nutr. 60, 1783–1796. 10.1080/10408398.2019.1599812 31062600

[B3] Banks W. A. (2006). The blood–brain barrier as a regulatory interface in the gut–brain axes. Physiol. Behav. 89, 472–476. 10.1016/j.physbeh.2006.07.004 16904139

[B4] Boef A. G. Dekkers O. M. le Cessie S. (2015). Mendelian randomization studies: a review of the approaches used and the quality of reporting. Int. J. Epidemiol. 44, 496–511. 10.1093/ije/dyv071 25953784

[B5] Bowden J. Davey Smith G. Burgess S. (2015). Mendelian randomization with invalid instruments: effect estimation and bias detection through Egger regression. Int. J. Epidemiol. 44, 512–525. 10.1093/ije/dyv080 26050253 PMC4469799

[B6] Braniste V. Al-Asmakh M. Kowal C. Anuar F. Abbaspour A. Tót M.. (2014). The gut microbiota influences blood-brain barrier permeability in mice. Sci. Transl. Med. 6, 263ra158. 10.1126/scitranslmed.3009759 25411471 PMC4396848

[B7] Brion M. -J. A. Shakhbazov K. Visscher P. M. (2013). Calculating statistical power in Mendelian randomization studies. Int. J. Epidemiol. 42, 1497–1501. 10.1093/ije/dyt179 24159078 PMC3807619

[B8] Bui T. P. N. Shetty S. A. Lagkouvardos I. Ritari J. Chamlagain B. Douillard F. P.. (2016). Comparative genomics and physiology of the butyrate-producing bacterium Intestinimonas butyriciproducens. Environ. Microbiol. Rep. 8, 1024–1037. 10.1111/1758-2229.12483 27717172

[B9] Bui T. P. N. Troise A. D. Nijsse B. Roviello G. N. Fogliano V. de Vos W. M. (2020). Intestinimonas-like bacteria are important butyrate producers that utilize Nε-fructosyllysine and lysine in formula-fed infants and adults. J. Funct. Food. 70:103974. 10.1016/j.jff.2020.103974

[B10] Burgess S. Butterworth A. Thompson S. G. (2013). Mendelian randomization analysis with multiple genetic variants using summarized data. Genet. Epidemiol. 37, 658–665. 10.1002/gepi.21758 24114802 PMC4377079

[B11] Burgess S. Dudbridge F. Thompson S. G. (2016). Combining information on multiple instrumental variables in Mendelian randomization: comparison of allele score and summarized data methods. Stat. Med. 35, 1880–1906. 10.1002/sim.6835 26661904 PMC4832315

[B12] Burgess S. Thompson S. G. (2015). Multivariable Mendelian randomization: the use of pleiotropic genetic variants to estimate causal effects. Am. J. Epidemiol. 181, 251–260. 10.1093/aje/kwu283 25632051 PMC4325677

[B13] Burgess S. Thompson S. G. (2017). Interpreting findings from Mendelian randomization using the MR-Egger method. Eur. J. Epidemiol. 32, 377–389. 10.1007/s10654-017-0255-x 28527048 PMC5506233

[B14] Chau J. K. Lin J. R. Atashband S. Irvine R. A. Westerberg B. D. (2010). Systematic review of the evidence for the etiology of adult sudden sensorineural hearing loss. Laryngoscope. 120, 1011–1021. 10.1002/lary.20873 20422698

[B15] Cui B. Su D. Li W. She X. Zhang M. Wang R.. (2018). Effects of chronic noise exposure on the microbiome-gut-brain axis in senescence-accelerated prone mice: implications for Alzheimer's disease. J. Neuroinflammation. 15, 190. 10.1186/s12974-018-1223-4 29933742 PMC6015475

[B16] Dalile B. Van Oudenhove L. Vervliet B. Verbeke K. (2019). The role of short-chain fatty acids in microbiota–gut–brain communication. Nat. Rev. Gastroenterol. Hepatol. 16, 461–478. 10.1038/s41575-019-0157-3 31123355

[B17] Davey Smith G. (2014). Mendelian randomization: genetic anchors for causal inference in epidemiological studies. Hum. Mol. Genet. 23, R89–R98. 10.1093/hmg/ddu328 25064373 PMC4170722

[B18] Del Greco M. F. Minelli C. Sheehan N. A. Thompson J. R. (2015). Detecting pleiotropy in Mendelian randomisation studies with summary data and a continuous outcome. Stat. Med. 34, 2926–2940. 10.1002/sim.6522 25950993

[B19] Denton A. J. Godur D. A. Mittal J. Bencie N. B. Mittal R. Eshraghi A. A. (2022). Recent advancements in understanding the gut microbiome and the inner ear axis. Otolaryngol. Clin. North Am. 55, 1125–1137. 10.1016/j.otc.2022.07.002 36088154

[B20] Fan J. Zhou Y. Meng R. Tang J. Zhu J. Aldrich M. C.. (2023). Cross-talks between gut microbiota and tobacco smoking: a two-sample Mendelian randomization study. BMC Med. 21, 163. 10.1186/s12916-023-02863-1 37118782 PMC10148467

[B21] Fortunato S. Forli F. Guglielmi V. De Corso E. Paludetti G. Berrettini S.. (2016). A review of new insights on the association between hearing loss and cognitive decline in ageing. Acta. Otorhinolaryngol. Ital. 36, 155–166. 10.14639/0392-100X-993 27214827 PMC4977003

[B22] Fousekis F. S. Saridi M. Albani E. Daniel F. Katsanos K. H. Kastanioudakis I. G.. (2018). Ear involvement in inflammatory bowel disease: a review of the literature. J. Clin. Med. Res. 10, 609–614. 10.14740/jocmr3465w 29977417 PMC6031254

[B23] Guo M. Li Z. (2019). Polysaccharides isolated from Nostoc commune Vaucher inhibit colitis-associated colon tumorigenesis in mice and modulate gut microbiota. J. Funct. Food. 10, 6873–6881. 10.1039/C9FO00296K 31584586

[B24] Hartwig F. P. Davey Smith G. Bowden J. (2017). Robust inference in summary data Mendelian randomization via the zero modal pleiotropy assumption. Int. J. Epidemiol. 46, 1985–1998. 10.1093/ije/dyx102 29040600 PMC5837715

[B25] Hemani G. Tilling K. Davey Smith G. (2017). Orienting the causal relationship between imprecisely measured traits using GWAS summary data. PLoS Genet. 13:e1007081. 10.1371/journal.pgen.1007081 29149188 PMC5711033

[B26] Hirose K. Hartsock J. J. Johnson S. Santi P. Salt A. N. (2014). Systemic lipopolysaccharide compromises the blood-labyrinth barrier and increases entry of serum fluorescein into the perilymph. J. Assoc. Res. Otolaryngol. 15, 707–719. 10.1007/s10162-014-0476-6 24952083 PMC4164684

[B27] Hung S. -C. Liao K. -F. Muo C. -H. Lai S. -W. Chang C. -W. Hung H. -C. (2015). Hearing loss is associated with risk of Alzheimer's disease: a case-control study in older people. J. Epidemiol. 25, 517–521. 10.2188/jea.JE20140147 25986155 PMC4517989

[B28] Ichimiya I. Yoshida K. Hirano T. Suzuki M. Mogi G. (2000). Significance of spiral ligament fibrocytes with cochlear inflammation. Int. J. Pediatr. Otorhinolaryngol. 56, 45–51. 10.1016/S0165-5876(00)00408-0 11074115

[B29] Juhn S. K. Hunter B. A. Odland R. M. (2001). Blood-labyrinth barrier and fluid dynamics of the inner ear. Int. Tinnitus. J. 7, 72–83. 14689642

[B30] Kalyoncu D. Urganci N. Calis A. B. Ozbal A. (2010). Sensorineural hearing loss in pediatric patients with inflammatory bowel disease. Dig. Dis. Sci. 55, 150–152. 10.1007/s10620-009-0714-7 19255853

[B31] Karmody C. S. Valdez T. A. Desai U. Blevins N. H. (2009). Sensorineural hearing loss in patients with inflammatory bowel disease. Am. J. Otolaryngol. 30, 166–170. 10.1016/j.amjoto.2008.04.009 19410121

[B32] Kociszewska D. Chan J. Thorne P. R. Vlajkovic S. M. (2021). The link between gut dysbiosis caused by a high-fat diet and hearing loss. Int. J. Mol. Sci. 22:13177. 10.3390/ijms222413177 34947974 PMC8708400

[B33] Kociszewska D. Vlajkovic S. (2022a). Age-related hearing loss: the link between inflammaging, immunosenescence, and gut dysbiosis. Int. J. Mol. Sci. 23:7348. 10.3390/ijms23137348 35806352 PMC9266910

[B34] Kociszewska D. Vlajkovic S. M. (2022b). The association of inflammatory gut diseases with neuroinflammatory and auditory disorders. Front. Biosci. 14:8. 10.31083/j.fbe1402008 35730449

[B35] Kuhn M. Heman-Ackah S. E. Shaikh J. A. Roehm P. C. (2011). Sudden sensorineural hearing loss: a review of diagnosis, treatment, and prognosis. Trends. Amplif. 15, 91–105. 10.1177/1084713811408349 21606048 PMC4040829

[B36] Kurilshikov A. Medina-Gomez C. Bacigalupe R. Radjabzadeh D. Wang J. Demirkan A.. (2021). Large-scale association analyses identify host factors influencing human gut microbiome composition. Nat. Genet. 53, 156–165. 10.1038/s41588-020-00763-1 33462485 PMC8515199

[B37] Kurki M. I. Karjalainen J. Palta P. Sipilä T. P. Kristiansson K. Donner K.. (2022). FinnGen: unique genetic insights from combining isolated population and national health register data. medRxiv. 10.1101/2022.03.03.22271360

[B38] Li P. Wang H. Guo L. Gou X. Chen G. Lin D.. (2022). Association between gut microbiota and preeclampsia-eclampsia: a two-sample Mendelian randomization study. BMC Med. 20, 443. 10.1186/s12916-022-02657-x 36380372 PMC9667679

[B39] Lin Z. Deng Y. Pan W. (2021). Combining the strengths of inverse-variance weighting and Egger regression in Mendelian randomization using a mixture of regressions model. PLoS Genet. 17:e1009922. 10.1371/journal.pgen.1009922 34793444 PMC8639093

[B40] Liu K. Wu P. Zou J. Fan H. Hu H. Cheng Y.. (2022). Mendelian randomization analysis reveals causal relationships between gut microbiome and optic neuritis. Hum. Genet. 142, 1139–1148. 10.1007/s00439-022-02514-0 36576600

[B41] Liu X. Cheng Y. -W. Shao L. Sun S. -H. Wu J. Song Q. -H.. (2021). Gut microbiota dysbiosis in Chinese children with type 1 diabetes mellitus: an observational study. World J. Gastroenterol. 27, 2394–2414. 10.3748/wjg.v27.i19.2394 34040330 PMC8130045

[B42] Ma L. Ni Y. Wang Z. Tu W. Ni L. Zhuge F.. (2020). Spermidine improves gut barrier integrity and gut microbiota function in diet-induced obese mice. Gut. Microbes. 12, 1–19. 10.1080/19490976.2020.1832857 33151120 PMC7668533

[B43] Mujica-Mota M. A. Patel N. Saliba I. (2018). Hearing loss in type 1 diabetes: are we facing another microvascular disease? a meta-analysis. Int. J. Pediatr. Otorhinolaryngol. 113, 38–45. 10.1016/j.ijporl.2018.07.005 30174007

[B44] O'Connor L. J. Price A. L. (2018). Distinguishing genetic correlation from causation across 52 diseases and complex traits. Nat. Genet. 50, 1728–1734. 10.1038/s41588-018-0255-0 30374074 PMC6684375

[B45] Ong J. S. MacGregor S. (2019). Implementing MR-PRESSO and GCTA-GSMR for pleiotropy assessment in Mendelian randomization studies from a practitioner's perspective. Genet. Epidemiol. 43, 609–616. 10.1002/gepi.22207 31045282 PMC6767464

[B46] Reay W. R. Kiltschewskij D. J. Geaghan M. P. Atkins J. R. Carr V. J. Green M. J.. (2022). Genetic estimates of correlation and causality between blood-based biomarkers and psychiatric disorders. Sci. Adv. 8:eabj8969. 10.1126/sciadv.abj8969 35385317 PMC8986101

[B47] Richmond R. C. George Davey Smith G. D. (2022). Mendelian randomization: concepts and scope. Cold Spring Harb. Perspect. Med. 12:a040501. 10.1101/cshperspect.a040501 34426474 PMC8725623

[B48] Rinninella E. Raoul P. Cintoni M. Franceschi F. Miggiano G. A. D. Gasbarrini A.. (2019). What is the healthy gut microbiota composition? a changing ecosystem across age, environment, diet, and diseases. Microorganisms. 7:14. 10.3390/microorganisms7010014 30634578 PMC6351938

[B49] Sanna S. van Zuydam N. R. Mahajan A. Kurilshikov A. Vila A. V. Võsa U.. (2019). Causal relationships among the gut microbiome, short-chain fatty acids and metabolic diseases. Nat. Genet. 51, 600–605. 10.1038/s41588-019-0350-x 30778224 PMC6441384

[B50] Sekula P. Del Greco M. F Pattaro C. Köttgen A. (2016). Mendelian randomization as an approach to assess causality using observational data. J. Am. Soc. Nephrol. 27, 3253–3265. 10.1681/ASN.2016010098 27486138 PMC5084898

[B51] Shen Y. Hu H. Fan C. Wang Q. Zou T. Ye B.. (2021). Sensorineural hearing loss may lead to dementia-related pathological changes in hippocampal neurons. Neurobiol. Dis. 156:105408. 10.1016/j.nbd.2021.105408 34082124

[B52] Song C. I. Pogson J. M. Andresen N. S. Ward B. K. (2021). Mri with gadolinium as a measure of blood-labyrinth barrier integrity in patients with inner ear symptoms: a scoping review. Front. Neurol. 12:662264. 10.3389/fneur.2021.662264 34093410 PMC8173087

[B53] Staiger D. O. Stock J. H. (1994). Instrumental Variables Regression With Weak Instruments. National Bureau of Economic Research Cambridge. 10.3386/t0151

[B54] Storey J. D. Tibshirani R. (2003). Statistical significance for genomewide studies. Proc. Natl. Acad. Sci. U. S. A. 100, 9440–9445. 10.1073/pnas.1530509100 12883005 PMC170937

[B55] Sun L. Jia H. Li J. Yu M. Yang Y. Tian D.. (2019). Cecal gut microbiota and metabolites might contribute to the severity of acute myocardial ischemia by impacting the intestinal permeability, oxidative stress, and energy metabolism. Front. Microbiol. 10:1745. 10.3389/fmicb.2019.01745 31428065 PMC6687875

[B56] Tang T. -H. Hwang J. -H. Yang T. -H. Hsu C. -J. Wu C. -C. Liu T. -C. (2019). Can nutritional intervention for obesity and comorbidities slow down age-related hearing impairment? Nutrients. 11:1668. 10.3390/nu11071668 31330876 PMC6682960

[B57] Tanna R. J. Lin J. W. de Jesus O. (2020). Sensorineural Hearing Loss. 33351419

[B58] Tarshish Y. Leschinski A. Kenna M. (2013). Pediatric sudden sensorineural hearing loss: diagnosed causes and response to intervention. Int. J. Pediatr. Otorhinolaryngol. 77, 553–559. 10.1016/j.ijporl.2013.01.004 23369615

[B59] Wengrower D. Koslowsky B. Peleg U. Mazuz B. Cohen L. Ben-David A.. (2016). Hearing loss in patients with inflammatory bowel disease. Dig. Dis. Sci. 61, 2027–2032. 10.1007/s10620-016-4074-9 27048450

[B60] World Health Organization (2021). World Report on Hearing. World Health Organization.

[B61] Xu Q. Ni J. -J. Han B. -X. Yan S. -S. Wei X. -T. Feng G. -J.. (2022). Causal relationship between gut microbiota and autoimmune diseases: a two-sample Mendelian randomization study. Front. Immunol. 12:5819. 10.3389/fimmu.2021.746998 35140703 PMC8819003

[B62] Xue H. Shen X. Pan W. (2021). Constrained maximum likelihood-based Mendelian randomization robust to both correlated and uncorrelated pleiotropic effects. Am. J. Hum. Genet. 108, 1251–1269. 10.1016/j.ajhg.2021.05.014 34214446 PMC8322939

[B63] Yu H. Wan X. Yang M. Xie J. Xu K. Wang J.. (2023). A large-scale causal analysis of gut microbiota and delirium: a Mendelian randomization study. J. Affect Disord. 329, 64–71. 10.1016/j.jad.2023.02.078 36842654

